# Design and Fabrication of Millimeter-Wave Frequency-Tunable Metamaterial Absorber Using MEMS Cantilever Actuators

**DOI:** 10.3390/mi13081354

**Published:** 2022-08-20

**Authors:** Myungjin Chung, Heijun Jeong, Yong-Kweon Kim, Sungjoon Lim, Chang-Wook Baek

**Affiliations:** 1 Department of Electrical and Computer Engineering, Seoul National University, 1 Gwanak-ro, Gwanak-gu, Seoul 08826, Korea; 2 School of Electrical and Electronics Engineering, Chung-Ang University, 84 Heukseok-ro, Dongjak-gu, Seoul 06974, Korea

**Keywords:** millimeter wave, frequency tunable, metamaterial absorber, MEMS cantilever, stress-gradient, plasma ashing

## Abstract

In this paper, a MEMS (Micro Electro Mechanical Systems)-based frequency-tunable metamaterial absorber for millimeter-wave application was demonstrated. To achieve the resonant-frequency tunability of the absorber, the unit cell of the proposed metamaterial was designed to be a symmetric split-ring resonator with a stress-induced MEMS cantilever array having initial out-of-plane deflections, and the cantilevers were electrostatically actuated to generate a capacitance change. The dimensional parameters of the absorber were determined via impedance matching using a full electromagnetic simulation. The designed absorber was fabricated on a glass wafer with surface micromachining processes using a photoresist sacrificial layer and the oxygen-plasma-ashing process to release the cantilevers. The performance of the fabricated absorber was experimentally validated using a waveguide measurement setup. The absorption frequency shifted down according to the applied DC (direct current) bias voltage from 28 GHz in the initial off state to 25.5 GHz in the pull-down state with the applied voltage of 15 V. The measured reflection coefficients at those frequencies were −5.68 dB and −33.60 dB, corresponding to the peak absorptivity rates of 72.9 and 99.9%, respectively.

## 1. Introduction

Metamaterials, artificially engineered composites of periodic or non-periodic structure, show a peculiar and exotic electromagnetic behavior, unlike materials found in nature [1]. Thanks to their discovery, many studies on the intriguing phenomena related to metamaterials have been published, such as metalens [2,3], slow light [4], chirality [5], materials with a negative refractive index [6], those capable of perfect absorption [7], etc. In particular, metamaterial absorbers, due to their unique characteristics and flexibility, have been applied for various purposes, such as thermal emitters [8], chemical sensors [9], imaging [10], optical cloaking [11], etc. Compared with the conventional electromagnetic absorbers, such as wedge-tapered [12] or Jaumann [13] absorbers, which are bulky and have high material costs, metamaterial absorbers can accomplish high absorptivity with a very thin structure using low-cost fabrication processes. The absorption properties can be achieved by controlling the permittivity and permeability by specially designing the geometry of the unit cell of the metamaterials. Therefore, many studies on metamaterial absorbers have been conducted to design these unit cells to fit the target frequency, e.g., millimeter wave [14,15], THz regime [16,17], and infrared [18] regime. Many of these works have demonstrated remarkable performance in term of absorptivity and simplicity in the fabrication processes. However, due to their inherent resonant characteristics, metamaterial absorbers have the limitation of the narrow absorption bandwidth.

One of the ways to overcome this drawback is to actively reconfigure the resonant frequency of the metamaterial with tunable elements. Some tuning methods have been reported to demonstrate frequency-reconfigurable metamaterial absorbers. Electrical tuning using varactor or PIN (positive-intrinsic-negative) diodes allows a fast tuning speed and a wide tuning range to be obtained [19,20,21], but high-frequency applications above 10 GHz are limited due to the performance degradation of the tunable elements. Material-based tuning methods using liquid crystals [22], phase-change materials (PCMs) [23,24], and graphene [25,26,27,28,29,30] have also shown a wide spectral range and a considerable tuning ratio, but they have suffered from temperature constraints and unusual fabrication difficulties. An intuitive and straightforward way for obtaining tunability is to reconfigure the shape of the unit cell structure of the metamaterial. We can change the resonant frequency of the absorber without the degradation of other device performances by mechanically deforming all or part of the unit cell structure. This mechanical reconfiguration method, however, faces miniaturization issues as the operating frequency increases. For this reason, MEMS-based tunable metamaterial absorbers, in which micromechanical actuators are used to reconfigure the metamaterial, have been reported for higher frequencies [31,32,33]. Most of the studies reported at present are focused on the THz/sub-THz regime, because the scales of the MEMS fabrication processes are suitable for those frequency ranges. On the contrary, it is difficult to find MEMS-based tunable absorbers working at millimeter-wave frequencies. The increase in the driving voltage and power consumption to obtain a noticeable amount of frequency reconfiguration, fabrication nonuniformity, and difficulty in the construction of the absorber measurement system at millimeter-wave frequencies could be the possible reasons.

In this paper, a frequency-tunable metamaterial absorber targeting millimeter-wave frequency (Ka-band) applications using MEMS cantilever actuators was designed. Electrostatically driven metal MEMS cantilevers were implemented in the split-ring resonators (SRRs) of the metamaterial unit cell as variable capacitors to reconfigure the resonant frequency. In order to achieve a large capacitance change with a lower driving voltage, MEMS cantilevers were fabricated on the capacitive area in the form of an array structure with a bent-up curvature using stress gradients induced by the oxygen-plasma-release process of the cantilevers. The proposed absorber with two unit cells was designed through full-wave simulation, fabricated via surface micromachining processes on a glass wafer, and experimentally characterized using a relatively simple waveguide measurement setup. The effects of the nonuniform bending of the fabricated cantilevers on the operating voltage and absorption performances were considered using the measured profiles of the fabricated cantilever beams.

## 2. Operation Principle and Device Structure

In general, an incident electromagnetic wave is divided into reflection, transmission, and absorption. Thus, the absorbance ($A\left(\omega \right)$), reflectance ($\Gamma \left(\omega \right))$, and transmittance ($Τ\left(\omega \right)$) have the following relationship:

(1) $$A\left(\omega \right)=1-\Gamma \left(\omega \right)-Τ\left(\omega \right)$$

If we assume that $Τ\left(\omega \right)$ is equal to zero due to metal deposition on the backside of the absorber, the highest absorptivity can be achieved by minimizing $\Gamma \left(\omega \right)$. That is, the reflectance upon normal incidence can be simply expressed by the terms of the impedance of the free space (${Ζ}_{0}=377\;\Omega$) and the impedance of the absorber medium (${Ζ}_{M}$):

(2) $$\Gamma \left(\omega \right)=\frac{{Ζ}_{M}-Z_{0}}{{Ζ}_{M}+{Ζ}_{0}}$$

In addition, the impedance of the absorber medium can be simply written in the following form:

(3) $$Z_{M}=\sqrt{\frac{\mu_{0}\mu_{r}}{\varepsilon_{0}\varepsilon_{r}}}$$

where $\varepsilon_{0}$ and $\mu_{0}$ are the permittivity and permeability of the free space, and $\varepsilon_{r}$ and $\mu_{r}$ are the permittivity and permeability of the absorber, respectively. Again, for high absorptivity, minimizing $\Gamma \left(\omega \right)$ can be achieved via impedance matching (${Ζ}_{0}=Z_{M}$). Under this condition, the structure and the design parameters are constructed.

The reflectance and transmittance can be converted into the reflection coefficient (R) and the transmission coefficient (T) using the following equations, respectively:

(4) $$R\;\left[\mathrm{dB}\right]=10\log\left(\Gamma \left(\omega \right)\right)$$

(5) $$T\;\left[\mathrm{dB}\right]=10\log\left(Τ\left(\omega \right)\right)$$

Since the transmittance is assumed to be nearly zero, the absorptivity can be calculated from the reflection coefficient:

(6) $$A=100\left(1-10^{\frac{R}{10}}\right)\left[\%\right]$$

**Figure 1:**
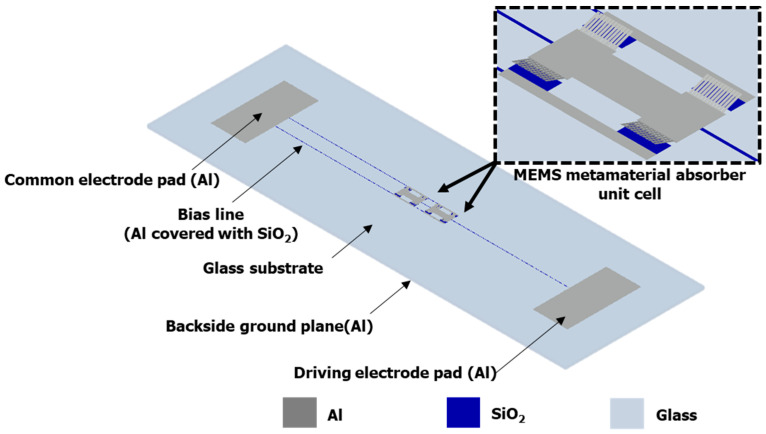
Three-dimensional schematic view of the proposed MEMS-driven frequency-tunable metamaterial absorber.

The entire schematic view of the proposed MEMS-driven frequency-tunable metamaterial absorber is shown in Figure 1. The absorber structure was designed to be characterized using a relatively simple waveguide measurement setup, which is described in Section 5. From the bottom to the top, the absorber consisted of an aluminum ground plane on the backside; a glass substrate; electrode pads/bias lines/driving electrodes, which were all made of aluminum; silicon dioxide layers on the driving electrodes and bias lines for electrical isolation; and two split-ring resonator (SRR) unit cells. Each SRR unit cell contained 4 sets of aluminum cantilever arrays, and each array consisted of 12 cantilevers. Each cantilever beam in the initial off state was bent up due to a process-induced stress gradient in the aluminum film. When a driving DC bias voltage was applied to the electrode pads, the beam moved downward to the on state because of the electrostatic force until it was completely pulled down to the bottom electrode. The angle that the MEMS cantilever beams made with the substrate decreased according to the bias voltage, and the resonant frequency shifted down from the initial off state to the on state due to the capacitance change that occurred due to the deflection of the cantilever beams.

**Figure 2:**
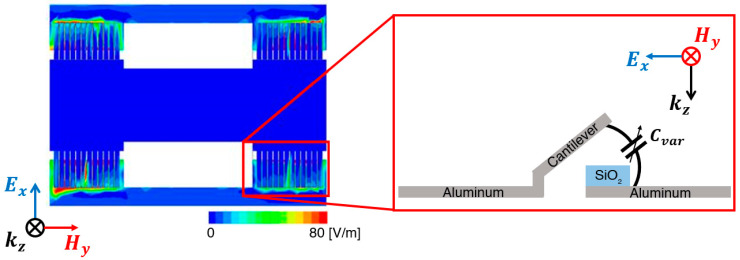
Electric-field distribution of the proposed metamaterial absorber unit cell.

Figure 2 shows the electric-field distribution of the proposed metamaterial absorber unit cell. The electric field was strongly distributed between the cantilever and metal part of the SRR on the substrate. Accordingly, the SRR capacitance could be regulated by changing the gap between the cantilever beam and the metal pattern of SRR underneath. As a result, frequency tunability could be realized with the following equation:

(7) $$f_{r}=\frac{1}{2\pi \sqrt{L\left(C+C_{v}\left(V\right)\right)}}$$

where $f_{r}$ is the resonant frequency, L is the inductance, C is the capacitance between the inner part of SRR and the outer part of the SRR, and $C_{v}\left(V\right)$ is the variable capacitance controlled by the cantilever array.

## 3. Design and Simulation

**Figure 3:**
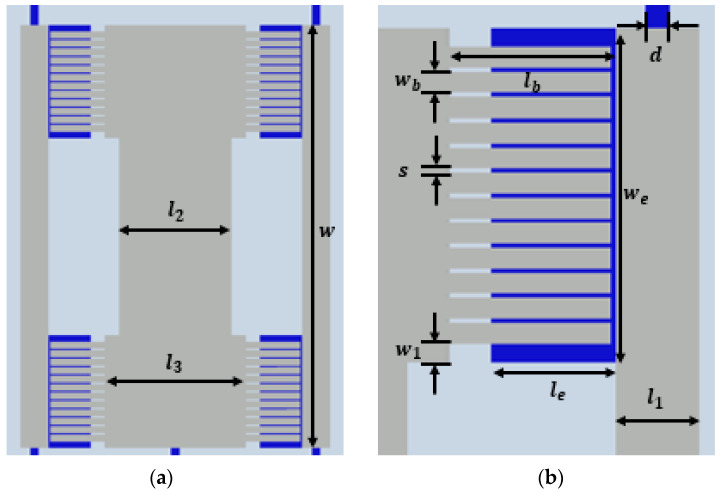

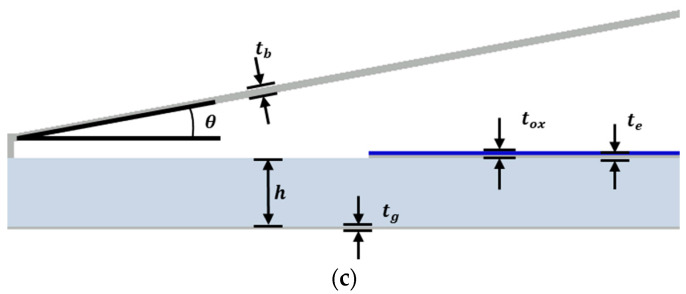
(**a**) Top view of the unit cell of the absorber. (**b**) Top view of the cantilever arrays in the unit cell. (**c**) Side view of the cantilever in the absorber.

To design and optimize the electromagnetic (EM) response of the proposed absorber, commercial full-wave EM simulation software (Ansys HFSS, R16) was used. The parameters of the proposed MEMS-based metamaterial absorber are shown in Figure 3. The detailed dimensions of the designed absorber are summarized in Table 1.

**Figure 4:**
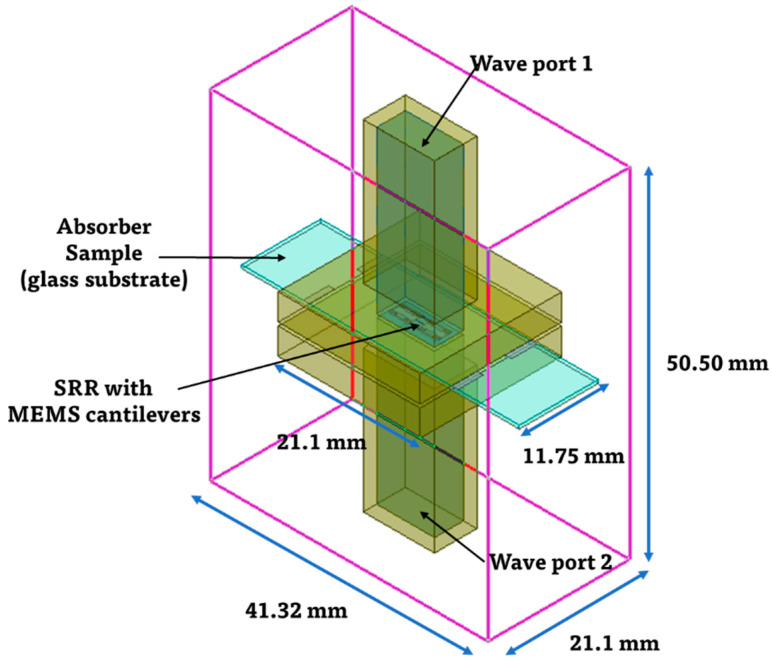
Electromagnetic simulation model including overall structures.

The simulation model including the overall structures was constructed by designing a pair of waveguides with the proposed absorber sample sandwiched between them, as shown in Figure 4. The waveguide dimensions were determined based on the size of the WR-34 waveguide (21.1 mm × 21.1 mm × 25.0 mm). The aluminum layer at the backside of the absorber was set to have a finite conductivity, and the radiation box was defined as an air material containing the waveguides and absorber sample, with the size of 41.32 mm × 21.10 mm × 50.50 mm. Two wave ports were set in the waveguide as the excitation port to excite the electromagnetic wave, as shown in Figure 4.

**Figure 5:**
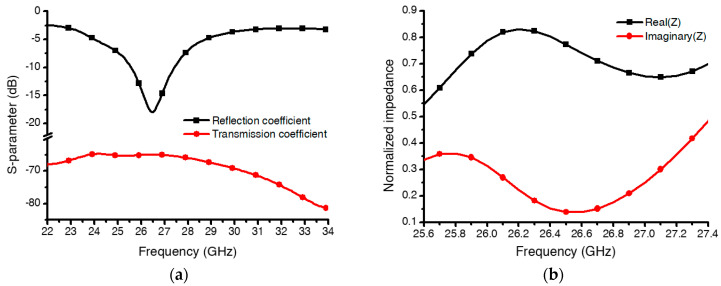

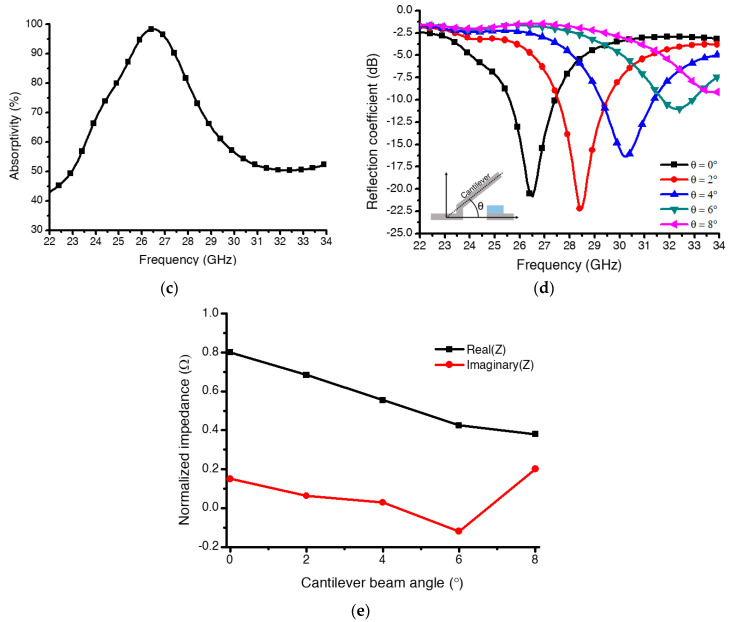
Analysis of the electromagnetic (EM) characteristics of the proposed metamaterial absorber: (**a**) reflection and transmission coefficients when the angle of the cantilever beams made with the substrate was *θ* = 0°; (**b**) normalized impedance from the free-space impedance of the proposed metamaterial absorber; (**c**) absorptivity calculated from the simulated S-parameter results; (**d**) reflection-coefficient results according to the variation in angle *θ* ; (**e**) normalized impedance according to the variation in angle *θ* at the resonant frequency.

The initial EM simulation results of the designed absorber are shown in Figure 5. As a substrate for the absorber, a borosilicate glass wafer (BOROFLOAT33; Shott AG, Mainz, Germany) was used, and its dielectric constant and loss tangent were assumed to be 4.2 and 0.07, respectively. For simplicity of the simulation, the MEMS cantilevers were assumed to be flat, straight beam structures instead of the curved structures that fabricated MEMS cantilevers beams would really be. Figure 5a shows the simulated reflection and transmission coefficient when the cantilever beams were in their on states, in which the beams were flat and parallel to the substrate. The absorber with these cantilever-beam structures had a reflection coefficient of −18 dB at 26.4 GHz. Since the bottom plane was totally covered with metallic ground, the transmission coefficient was below −60 dB. Accordingly, the normalized real and imaginary impedance ($Z_{M}/Z_{0}$) were 0.78 and 0.12, respectively, as shown in Figure 5b. As a result, the proposed metamaterials absorber had an absorptivity of 98.5% at 26.4 GHz, as shown in Figure 5c. To check the frequency tunability, the reflection coefficients were simulated according to tilting angle *θ* of the MEMS cantilever beam with respect to the substrate surface. As expected from Figure 5d, the absorption frequency was increased from 26.4 GHz to 28.4, 30.2, 32.3, and 33.5 GHz when the tilting angle was increased from 0° to 2°, 4°, 6°, and 8°, respectively. Therefore, the frequency tunability was confirmed via numerical simulation according to the cantilever-beam angle variation. In Figure 5d, we can see that the value of the reflection coefficient was gradually lowered as the tilting angle increased beyond 2° and then became lower than 10 dB when the angle reached 8°. Figure 5e shows the simulation result of the normalized impedance according to the variation in the cantilever-beam angles at the resonant frequency. As shown in Figure 5e, the real impedance gradually decreased from 0.8 to 0.4, and the imaginary impedance changed from a minimum value of −0.1 to a maximum value of 0.2, corresponding to variable cantilever-beam angles, because the effective permittivity and permeability varied according to the cantilever-beam angles. Therefore, we aimed to suppress the tilting angle of the cantilever beam below 8° during the fabrication process by engineering the induced stress gradient in the beam caused by the oxygen-plasma-release process.

## 4. Fabrication Process

**Figure 6:**
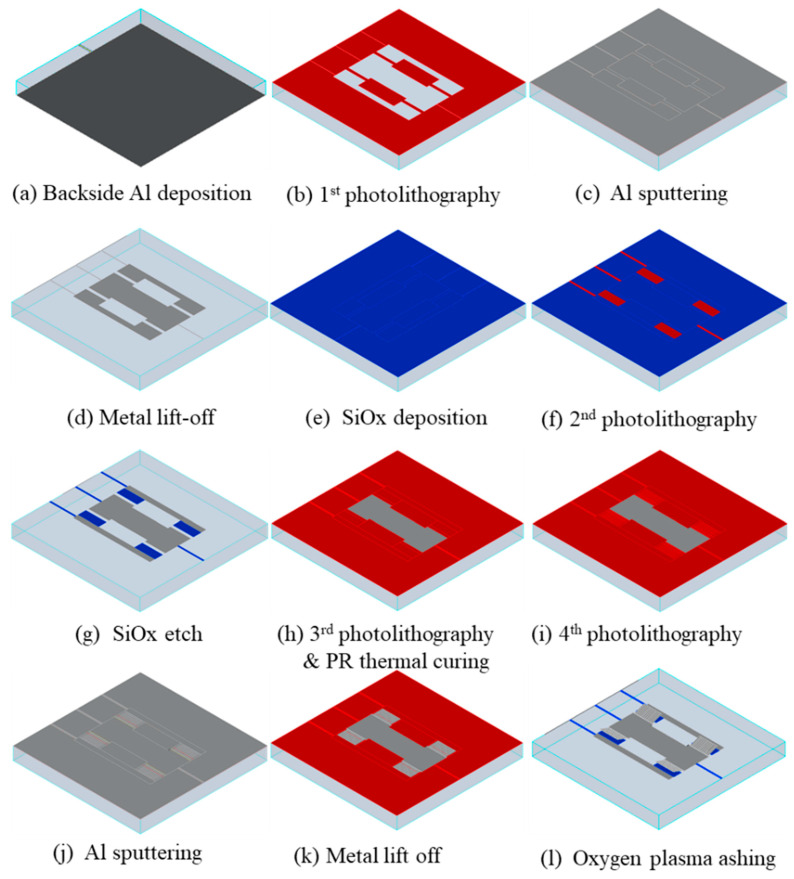
The overall fabrication process of the proposed absorber.

The overall fabrication process for the proposed absorber is illustrated in Figure 6. A 4 inch, 525 μm thick borosilicate glass wafer was used as the substrate for the absorber. The wafer was cleaned in a 4:1 mixture of sulfuric acid (H_2_SO_4_) and hydrogen peroxide (H_2_O_2_) to remove contaminants. On the backside of the wafer, a 300 nm thick aluminum layer was deposited by means of metal sputtering as a ground layer (Figure 6a). On the topside, common electrodes for the bias pads, driving electrodes, and bias lines were patterned via the metal sputtering of a 500 nm thick aluminum layer and a lift-off process using a negative photoresist (DNR-L300; Dongjin Semichem. Co., LTD. Seoul, Korea) (Figure 6b–d). Next, a 500 nm thick silicon-dioxide layer was deposited using plasma-enhanced chemical vapor deposition (PECVD) and patterned using the reactive-ion etching (RIE) process using CF_4_ gas to prevent electrical shorting between the MEMS cantilevers and the driving electrodes (Figure 6e–g). After that, a positive photoresist (AZ P4330-RS; Merck, Darmstadt, Germany) was spin-coated at the speed of 5000 rpm and patterned via a photolithography step as a sacrificial layer. The sacrificial layer was then thermally cured on the hot plate at the temperature of 210 °C for 1 h in order to make it stable for the subsequent processing steps (Figure 6h). The final thickness of the sacrificial layer after this thermal treatment was about 2.5 μm. Next, cantilever-beam arrays were patterned using aluminum sputtering and a lift-off process using a negative photoresist, again the DNR-L300 (Figure 6i,k). Finally, the oxygen plasma ashing of the sacrificial photoresist layer was used to release the cantilevers without stiction problems (Figure 6l). The ashing process was performed under an RF (radio frequency) power of 300 W, an oxygen flow rate of 150 sccm, and a chamber pressure of 100 Pa. It was previously reported that not only the stress created during the deposition process but also complicated factors during the plasma-ashing process may induce the deflection of the MEMS cantilevers [34,35]. To prevent the unintended, excessive bending of the cantilevers during plasma ashing, the asher chamber was sufficiently cooled down for more than a few hours after the first 10 min of etching; then another 10 min of etching followed.

**Figure 7:**
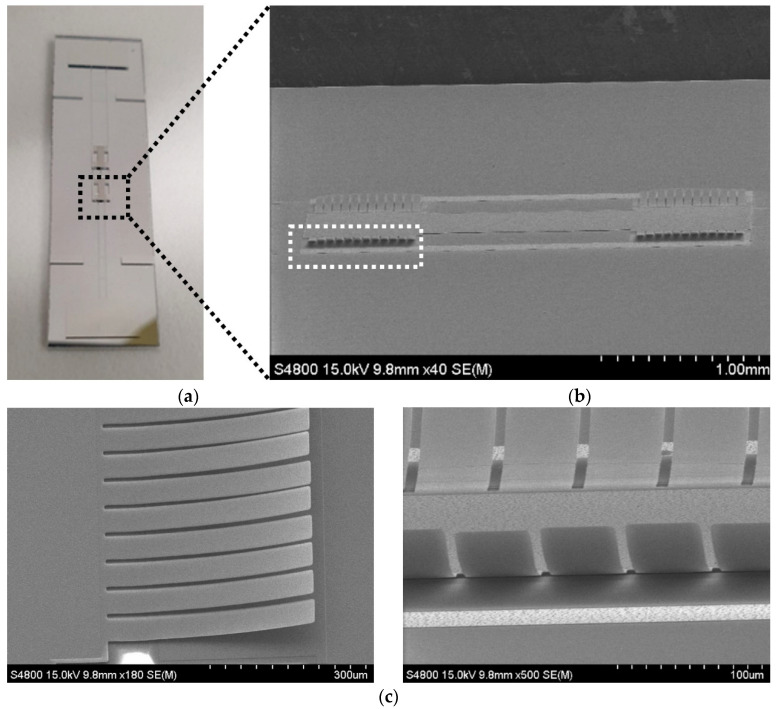
(**a**) Photograph of the fabricated absorber sample. (**b**) SEM image of the SRR unit cell. (**c**) Magnified SEM images of the released cantilever beams with stress gradients—side view (left) and end view (right).

Figure 7a shows a photograph of the fabricated absorber sample. The total sample size after dicing was 41.32 mm × 11.75 mm. Figure 7b shows the SEM (scanning electron microscopy) image of the SRR unit cell, and Figure 7c shows the magnified SEM image of an array structure of the released cantilever beams. As shown in Figure 7c, the fabricated cantilever beams were curved upwardly due to the stress variation in the thickness direction of the beam. Since the actual shape of the deformed cantilever was deviated from that of the simple inclined linear cantilever used in the initial simulation model, the electromechanical behavior of the cantilever beam was affected and eventually so were the EM characteristics of the fabricated absorber. The deformation profile of the cantilevers and its effects on the device were measured, and they are analyzed in the following sections.

## 5. Experimental Procedures and Results

### 5.1. Deformation Characteristics of the Released Cantilever Beams

**Figure 8:**
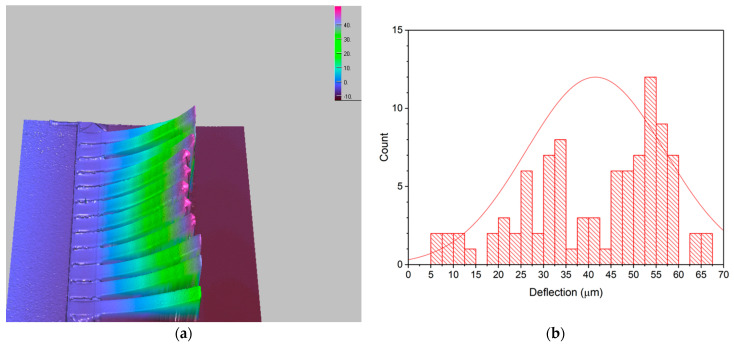
(**a**) Deformation profile image of 12 typical cantilever beams in the unit cell measured with a 3D optical surface profiler. (**b**) Distribution of the measured end-tip deflections for all the cantilevers in the fabricated sample.

As shown in the previous section, the released cantilever beams in the unit cell of the fabricated absorber were deformed upwardly with a certain radius of curvature, due to the induced stress gradient in the thickness direction of the beams. Residual stresses and stress gradients in the beam could depend not only on the deposition conditions but also on the post-deposition process. Theoretically, it was expected that all the cantilever beams in the sample fabricated throughout the same process flow had the same deformation profiles under the same stress gradients. The end-tip deflections and deformation profiles of the released cantilever beams, however, were measured to be different from each other, even in one set of arrays. The deformation profiles of the released cantilever beams of the absorber sample were measured with a 3D optical surface profiler (NanoFocus μSurf, NanoFocus AG, Oberhausen, Germany). The typical deformation plot of 12 cantilevers in one array of cantilevers from an SRR unit cell, for example, is shown in Figure 8a. In this array of cantilevers, the end-tip deflections varied from 31.16 to 59.33 μm. Therefore, the distribution of the end-tip deflections for all 96 cantilever beams in the fabricated sample was investigated, as shown in Figure 8b. The average of the end-tip deflections of the cantilevers was measured to be 41.5 μm, with a considerable amount of standard deviation of 15.4 μm. The measured average end-tip deflection corresponded to the tilting angle of about 5.9° if the cantilever beams were assumed to be straight.

The nonuniform bending of each cantilever beam in the sample might have been caused by various factors of the oxygen-plasma-ashing process we used to release the cantilever beams. It is known that heat is generated during the oxygen plasma ashing by decomposing the C_x_H_y_ bond into CO, CO_2_, and H_2_O via the reaction of active oxygen with the photoresist sacrificial layer. This heat effect can create the temperature gradient between the top and bottom surface of the beam structures, which can result in the permanent bending of the beams if it is too large. In addition, the microstructural change in the surface of the thin film exposed to plasma particles at a high energy level could be another factor leading to residual stress and thus the deflection of the cantilever beam [34].

### 5.2. Experimental Setup and Measurement Results

**Figure 9:**
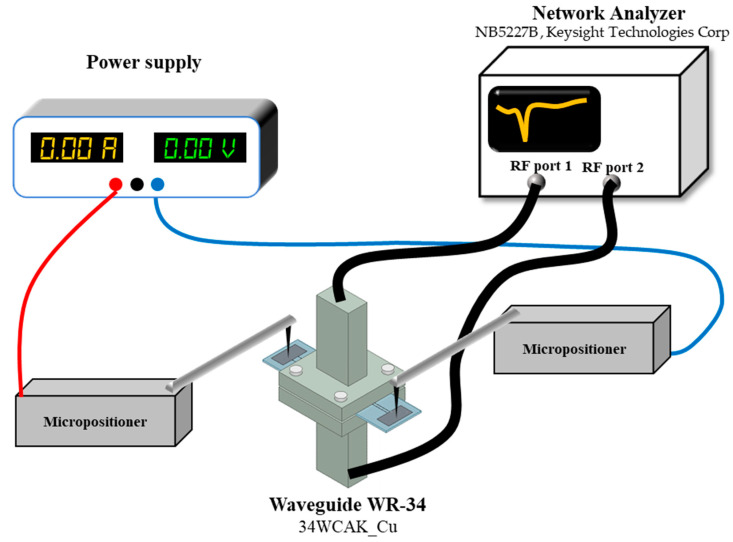
Schematic block diagram of the measurement setup.

In order to measure the absorption characteristics of the fabricated sample, a measurement setup was constructed, as shown in Figure 9. It employed an Agilent network analyzer (N5227B; Keysight Technologies, Santa Rosa, CA, USA) and two WR-34 waveguides (34WCAK_Cu; A-info, Irvine, CA, USA). The form factor of the waveguide was 8.636 mm × 4.318 mm, and the corresponding frequency range was from 22 GHz to 33 GHz. After the calibration of the network analyzer, the fabricated absorber sample was precisely aligned and embedded between the two waveguides. After the assembly process, two unit cells of the fabricated absorber were located inside the aperture of the waveguide, and the electrodes to actuate the MEMS cantilevers were exposed to the outside of the waveguide. Then, the probe tips of two micro-positioners (PB100; MS-TECH, Hwaseong, Korea) were made to contact with both the driving electrode and the common electrode, respectively, for applying a driving voltage to reconfigure the cantilever arrays. After connecting the micro-positioners to a power supply, a DC bias voltage was gradually applied to the electrodes of the absorber sample, and the reflection coefficients were measured in real time using the network analyzer. Only the reflection coefficients were measured to calculate absorptivity, because the backside of the absorber sample was completely covered with the deposited aluminum metal layer. The applied bias voltage was increased from zero to the voltage at which the reflection coefficient did not vary significantly.

**Figure 10:**
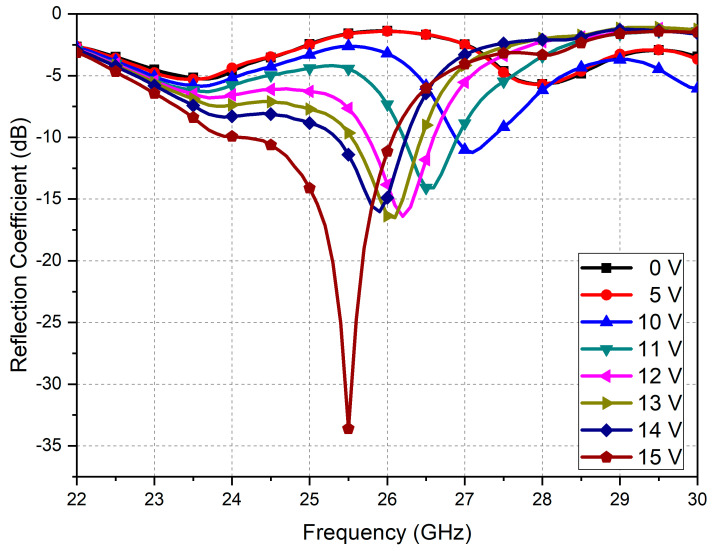
Measured reflection coefficients of the fabricated absorber sample according to the applied DC bias voltage ($V_{\mathrm{DC}}$).

The measured reflection coefficients of the fabricated absorber sample according to the applied DC bias voltages ($V_{\mathrm{DC}})$ are shown in Figure 10. In the initial state ($V_{\mathrm{DC}}=$ 0 V, i.e., all the MEMS cantilevers were deformed upward maximally), the resonant frequency was measured to be 28.0 GHz, with a reflection coefficient of −5.68 dB. Up to $V_{\mathrm{DC}}=$ 5 V, the reflection-coefficient curve did not vary significantly compared to the initial state. As the applied bias voltage further increased, the resonant frequency started to shift down to a lower value due to an increase in the capacitance via cantilever actuation, along with an increase in the reflection-coefficient value. An abrupt, large increase in the reflection coefficient was observed when $V_{\mathrm{DC}}=15$ V, at which all the MEMS cantilevers might have been pulled down, since no noticeable changes in the reflection-coefficient curve were observed for $V_{\mathrm{DC}}$ larger than 15 V. At this bias voltage of 15 V, the resonant frequency and the reflection coefficient were measured to be 25.5 GHz and −33.6 dB, respectively. The absorptivity could be calculated form the measured reflection coefficients using Equation (1) in Section 2. The measured resonant frequency, reflection/transmission coefficient, and calculated absorptivity at each applied bias voltage are summarized in Table 2. 

## 6. Discussion

In our initial simulation in Section 3, we assumed that the cantilever beams in the absorber were straight linear structures with the same tilting angles and that the angle could be varied from the maximum value to 0° without any limitation. The fabricated MEMS cantilever beams, however, had quadratic curvatures and nonuniform bending characteristics, which could in turn affect the mechanical behavior of the beam and the absorption properties according to the applied actuation voltage. Therefore, a direct comparison of the measured results in Figure 10 with the simulation results in Figure 5d was difficult. For this reason, some mechanical and EM simulations for the absorber were performed again reflecting the measured deflection profile of the fabricated cantilever beams.

**Figure 11:**
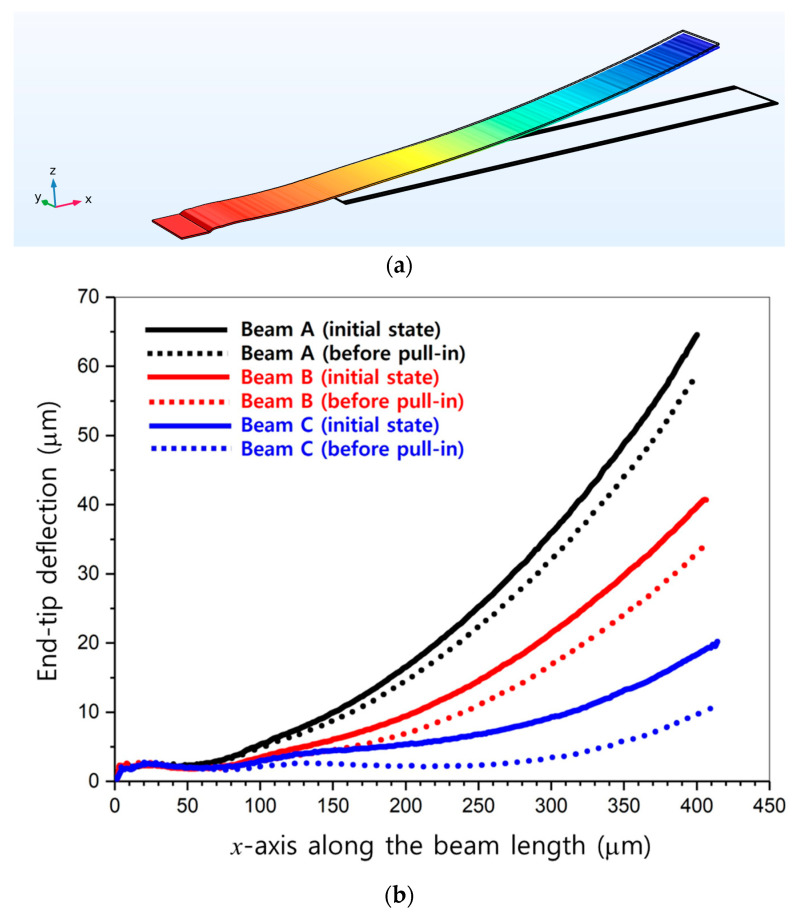
(**a**) COMSOL Multiphysics^®^ FEM model of the cantilever beam. (**b**) Initial deflection profiles of the three fabricated typical beams (solid lines, measured) and deflection profiles just prior to the pull-down state (dotted lines, simulated).

The typical finite element method (FEM) model of a single cantilever beam constructed using the measured profile is shown in Figure 11a. In addition to the beam curvature, a rounded and corrugated anchor shape resulting from the reflow of the photoresist sacrificial layer during the thermal curing process was also included in the modeling. These were clearly seen as solid lines representing the different initial deflection profiles of three fabricated beams, as shown in Figure 11b. Pull-in voltages for these three beams were simulated with COMSOL Multiphysics^®^ (version 6.0) and were estimated to be 24.6, 14.6, and 6.7 V for beams A, B, and C, respectively. In addition, the deformed profiles of these beams just before switching to the pull-down state were estimated with a COMSOL Multiphysics^®^ simulation and are illustrated as dotted lines in Figure 11b. It was observed that the amount of change in end-tip deflection we could obtain by varying the bias voltage was different for each beam, and it tended to become smaller for a beam with a larger initial deformation. It implied that the tilting angle of the fabricated beam that could be controlled by an electrostatic force was limited, as opposed to the simulation results in Figure 5d. For beam B with an initial end-tip deflection of 40.73 μm (i.e., close to the average deformation value), for example, the variation in the tilting angle we could achieve was limited from 5.81° to 4.86°. Therefore, if we assume that all beams in the absorber had the same uniform initial end-tip deflections, then the frequency shift would be small for the voltages before pull-down and suddenly become very large above the pull-down voltage. The measured reflection-coefficients data in Figure 10, however, showed a tendency of relatively continuous resonant-frequency change rather than a sudden pull-down behavior. This result was expected to some extent due to the fact that the fabricated beams in the measured sample had large and nonuniform initial deflection profiles.

**Figure 12:**
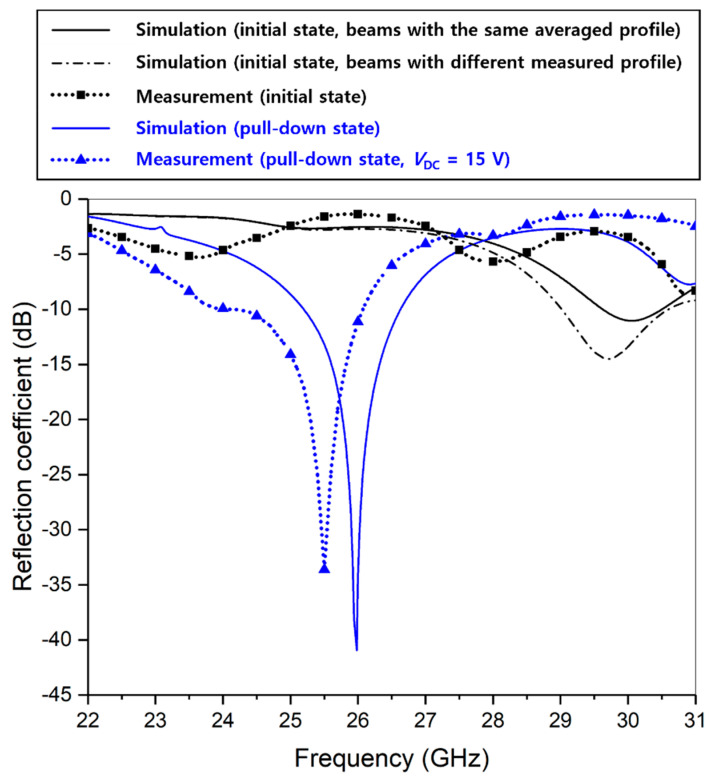
Comparison of the simulated reflection coefficients considering the initial deformation profiles of the fabricated beams with the measured reflection coefficients.

In Figure 12, the simulated reflection coefficients including the real variation in the initial deformation profiles of each fabricated cantilever beam are shown with the measurement results, both for the initial off state and pull-down on state. For comparison, the simulation result performed under the assumption that all the cantilevers had the same initial deformation profile of beam B in Figure 11 (i.e., the same average end-tip deflection of 41.5 μm) is also displayed. When we considered the effect of initial beam deformation, the simulated absorption frequency of the absorber in the initial state became lower and closer to the measured frequency range compared with the ideal simulation result in Figure 5d. The measured reflection coefficients in the pull-down state showed good agreement with the simulation results.

## 7. Conclusions

In this study, we proposed and demonstrated a MEMS-driven frequency-tunable metamaterial absorber operating at millimeter-wave frequencies. A surface-micromachined MEMS cantilever array as a part of the unit cell was fabricated to have process-induced stress gradients for a large tuning ratio with a relatively low actuation voltage. Both numerical and experimental demonstrations were performed on our metamaterial absorber. The absorptivity of the fabricated sample changed from 72.9% in the initial off state to 99.9% in the pull-down state, while the absorption frequency shifted from 28.0 to 25.5 GHz. The proposed MEMS-based metamaterial absorber could be employed to millimeter-wave applications where actively controlled electromagnetic absorbers are required but it is difficult to find effective tuning methods.

## Figures and Tables

**Table 1:** Dimensional parameters of the designed metamaterial absorber unit cell.

Symbols	Value	Description
*d*	40 μm	The width of the bias line
*l*_1_	200 μm	The edge length of the unit cell
*l*_2_	800 μm	The middle length of the anchor
*l*_3_	1000 μm	The length of the anchor
*l_e_*	300 μm	The length of the silicon oxide film
*l_b_*	400 μm	The length of the cantilever beam
*w*	3 mm	The width of the unit cell
*w*_1_	45 μm	The margin of the array edge
*w_b_*	50 μm	The width of the cantilever beams
*s*	10 μm	The space between cantilever beams
*t_b_*	1000 nm	The thickness of the cantilever beam
*t_ox_*	500 nm	The thickness of the silicon oxide
*t_e_*	500 nm	The thickness of the bottom electrode
*t_g_*	200 nm	The thickness of the Al ground plane
*h*	500 μm	The thickness of the glass substrate

**Table 2:** Summary of the measurement results.

Applied Voltage	Resonant Frequency	Reflection Coefficients	Absorptivity
0 V	28.0 GHz	−5.68 dB	72.9 %
5 V	27.9 GHz	−5.70 dB	73.0 %
10 V	27.1 GHz	−11.23 dB	92.5 %
11 V	26.6 GHz	−14.08 dB	96.1 %
12 V	26.2 GHz	−16.40 dB	97.7 %
13 V	26.1 GHz	−16.50 dB	97.8 %
14 V	25.9 GHz	−16.02dB	97.5 %
15 V	25.5 GHz	−33.60dB	99.9 %

## Data Availability

The data are available within the article.
